# Detonation nanodiamonds biofunctionalization and immobilization to titanium alloy surfaces as first steps towards medical application

**DOI:** 10.3762/bjoc.10.293

**Published:** 2014-11-26

**Authors:** Juliana P L Gonçalves, Afnan Q Shaikh, Manuela Reitzig, Daria A Kovalenko, Jan Michael, René Beutner, Gianaurelio Cuniberti, Dieter Scharnweber, Jörg Opitz

**Affiliations:** 1 Inspection and Diagnosis Methods, Fraunhofer Institute for Ceramic Technologies and Systems –Materials Diagnostics, Maria-Reiche-Str. 2, 01109 Dresden, Germany; 2 Max Bergmann Center of Biomaterials MBC, Technische Universität Dresden, Budapester Str. 27, 01069 Dresden, Germany; 3 Chair of General Biochemistry, Technische Universität Dresden, Bergstr. 66, 01069 Dresden, Germany

**Keywords:** biofunctionalization, carbon-nanomaterials, detonation nanodiamond, electrochemical immobilization, surface modification, titanium alloy

## Abstract

Due to their outstanding properties nanodiamonds are a promising nanoscale material in various applications such as microelectronics, polishing, optical monitoring, medicine and biotechnology. Beyond the typical diamond characteristics like extreme hardness or high thermal conductivity, they have additional benefits as intrinsic fluorescence due to lattice defects without photobleaching, obtained during the high pressure high temperature process. Further the carbon surface and its various functional groups in consequence of the synthesis, facilitate additional chemical and biological modification. In this work we present our recent results on chemical modification of the nanodiamond surface with phosphate groups and their electrochemically assisted immobilization on titanium-based materials to increase adhesion at biomaterial surfaces. The starting material is detonation nanodiamond, which exhibits a heterogeneous surface due to the functional groups resulting from the nitrogen-rich explosives and the subsequent purification steps after detonation synthesis. Nanodiamond surfaces are chemically homogenized before proceeding with further functionalization. Suspensions of resulting surface-modified nanodiamonds are applied to the titanium alloy surfaces and the nanodiamonds subsequently fixed by electrochemical immobilization. Titanium and its alloys have been widely used in bone and dental implants for being a metal that is biocompatible with body tissues and able to bind with adjacent bone during healing. In order to improve titanium material properties towards biomedical applications the authors aim to increase adhesion to bone material by incorporating nanodiamonds into the implant surface, namely the anodically grown titanium dioxide layer. Differently functionalized nanodiamonds are characterized by infrared spectroscopy and the modified titanium alloys surfaces by scanning and transmission electron microscopy. The process described shows an adsorption and immobilization of modified nanodiamonds on titanium; where aminosilanized nanodiamonds coupled with *O*-phosphorylethanolamine show a homogeneous interaction with the titanium substrate.

## Introduction

Detonation nanodiamonds (DND) are a promising carbon-derived nanoscale material, which has been investigated since some decades due to its outstanding properties, like extreme hardness and high thermal conductivity [1–3]. Beyond this, lattice defects, predominantly originating from nitrogen vacancy centers [4–5], enable fluorescence without photobleaching. Nanodiamonds can thus be used as markers for optical monitoring [6–7]. Toxicity has not been observed so far, so it has gained particular interest in biological and medical applications [8–9]. A number of publications on single cell labeling with nanodiamond particles [10–12] and use in therapeutic delivery were reported [13–15]. In such way, the low or non-existent toxicity and improved biocompatibility, in comparison to the unmodified DND, extends the applications of this carbon material from microelectronics, polishing and optical monitoring to biomedicine.

Titanium and titanium-based alloys have been used as bone implant material for a long time, because of their excellent corrosion properties, good mechanical strength and osteointegration, due to naturally occurring oxide layer at their surface [16]. Based on the surface oxide properties, various methods of surface modification of titanium and its alloys have been realized in the past decades to achieve improved surface properties with enhanced wear behavior, corrosion resistance, and biocompatibility.

Scharnweber et al. worked on biofunctionalization of titanium implant materials by immobilizing collagen type I and studied conformational changes caused by adsorptive immobilization [17–18]. Others worked on collagenous matrix coatings on titanium implants modified with decorin and chondroitin sulfate that enhanced osteoblast adhesion and increased expression of osteopontin (a bone-specific marker) [19]. Immobilization of biologically active molecules, by using tripartite molecules (linker, spacer and bioactive ligand), enables an easier process control and operability under nearly physiological conditions [20]. Scharnweber, Schwenzer and co-workers contributed to the development of implant materials based on titanium by demonstrating a new modular method of immobilization via regioselective partial incorporation of biofunctional molecules into anodically grown oxide layers. The authors electrochemically immobilized nucleic acids on titanium alloys for its surface modification with bioactive molecules [21–25], as, e.g., oligonucleotides and RGD peptide conjugates, for osteoblasts adhesion [26].

A successful incorporation of DND has already been achieved in a related research of our group in Dresden on nanodiamonds for corrosion protection and devising a nondestructive surveillance tool for corrosion monitoring in light metals such as aluminum and its alloys for aircraft industry [27].

Recently, the properties of nanodiamonds have been investigated in the field of bone tissue engineering [28–33]. For the presented study, a new hybrid implant material based on titanium alloy with functionalized nanodiamonds on anodic oxide layers is being conceived, to improve material properties. The motivation of our research was to perform first steps towards immobilization of functionalized DND to reinforce the surface of the material and to improve the exquisite properties of titanium to shape an improved material for medical uses.

**Figure 1:**
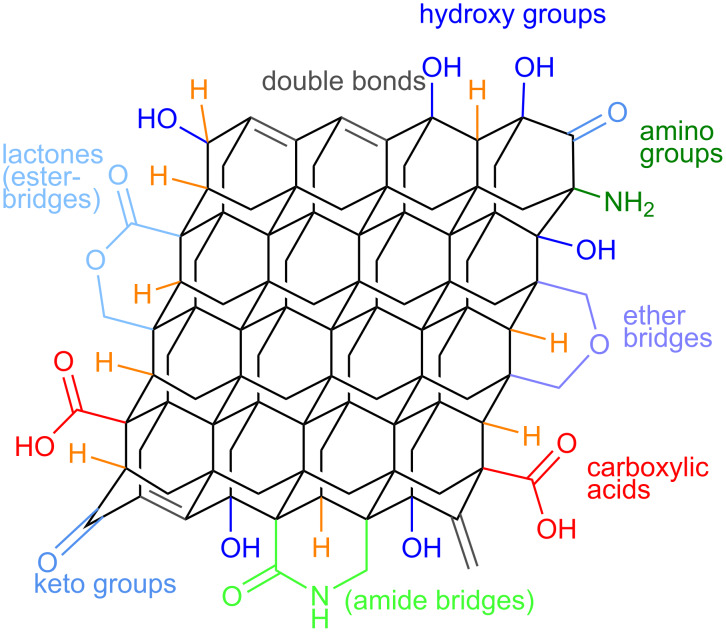
Proposed functional groups on purified DND surface.

DND exhibit several functional groups around the carbon core as shown in Figure 1, originating from the purification after the detonation process [28]. This enables task-oriented chemical or biological functionalization. For binding substances to titanium surfaces, phosphate groups are favored as anchors, because they possess a hydrolytically stable structure and support the formation of monolayers on metal oxide surfaces. [34–36] Especially in the case of titanium mono-, bi- or even tridentate phosphate coordination leads to exceptional strong binding that could not be achieved by mere electrostatic interaction or hydrogen bridging.

In this work we studied various functionalization methods for the attachment of the phosphate moieties to DND particles. We investigated further the binding of the modified nanodiamond particles to the surface of titanium alloys in combination with the formation of anodic oxide layers.

## Results and Discussion

### Covalent surface modification of DND

**Scheme 1:**
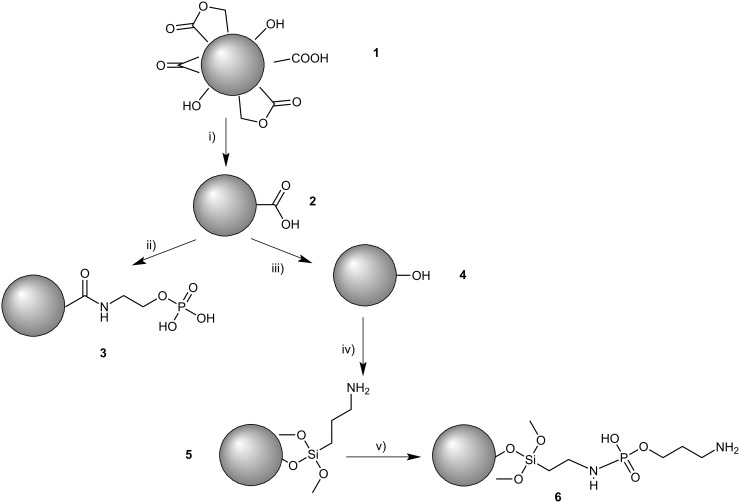
Overview on applied modification techniques to obtain DND with phosphate groups; conditions and reagents: (i) Sulfuric acid and nitric acid solution (9:1), 80 °C, 24 h (ii) *O*-phosphorylethanolamine (*O-*PEA), 1-ethyl-3-(3-dimethylaminopropyl)carbodiimide (EDC), 2-(*N*-morpholinoethanesulfonic acid), room temperature, 2.5 h; (iii) Sodium borohydride, ethanol, 60 °C, 24 h, (iv) (3-aminopropyltrimethoxysilane (APTMS), acidic environment, room temperature, 48 h, (v) *O-*PEA, EDC, 4-(dimethylamino)pyridine, dichloromethane (DCM), 0 °C → room temperature, 65 h.

The functionalization of nanodiamonds with phosphate groups was carried out considering protocols available in literature [37–39]. Those protocols were adapted and are summarized in Scheme 1.

Reaction step (ii) was performed to directly graft the phosphate group on the surface of the DND, whereas reactions (iii) and (iv) were performed to provide an amino group in the end of the functionalization, allowing the integration of further species, as shown in reaction (v). Even though product **5** does not contain a phosphate group, it was included as control to verify the interaction of amino groups and the titanium based alloy. Furthermore, products **3** and **6** can be seen as ‘inversely modified’ DND with a terminal phosphate and a secondary amide **3** or with a primary amine and a secondary phosphoroamidate **6**. This might help to assess the character of binding of amino and phosphate groups. The obtained products were characterized by infrared spectroscopy.

**Figure 2:**
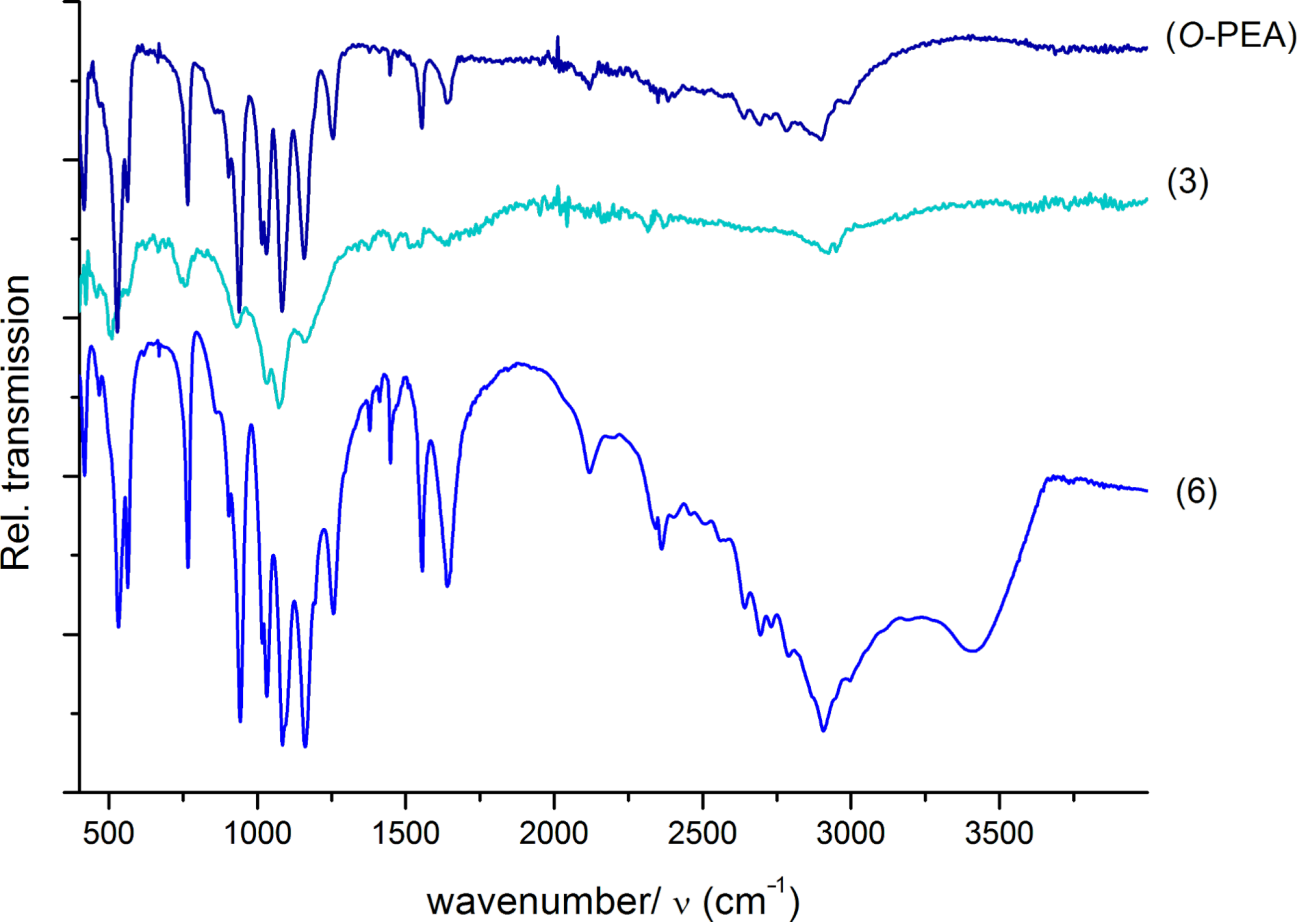
ATR-FTIR spectra of species **3** and **6** from Scheme 1 in comparison to *O*-phosphorylethanolamine (*O-*PEA).

**Figure 3:**
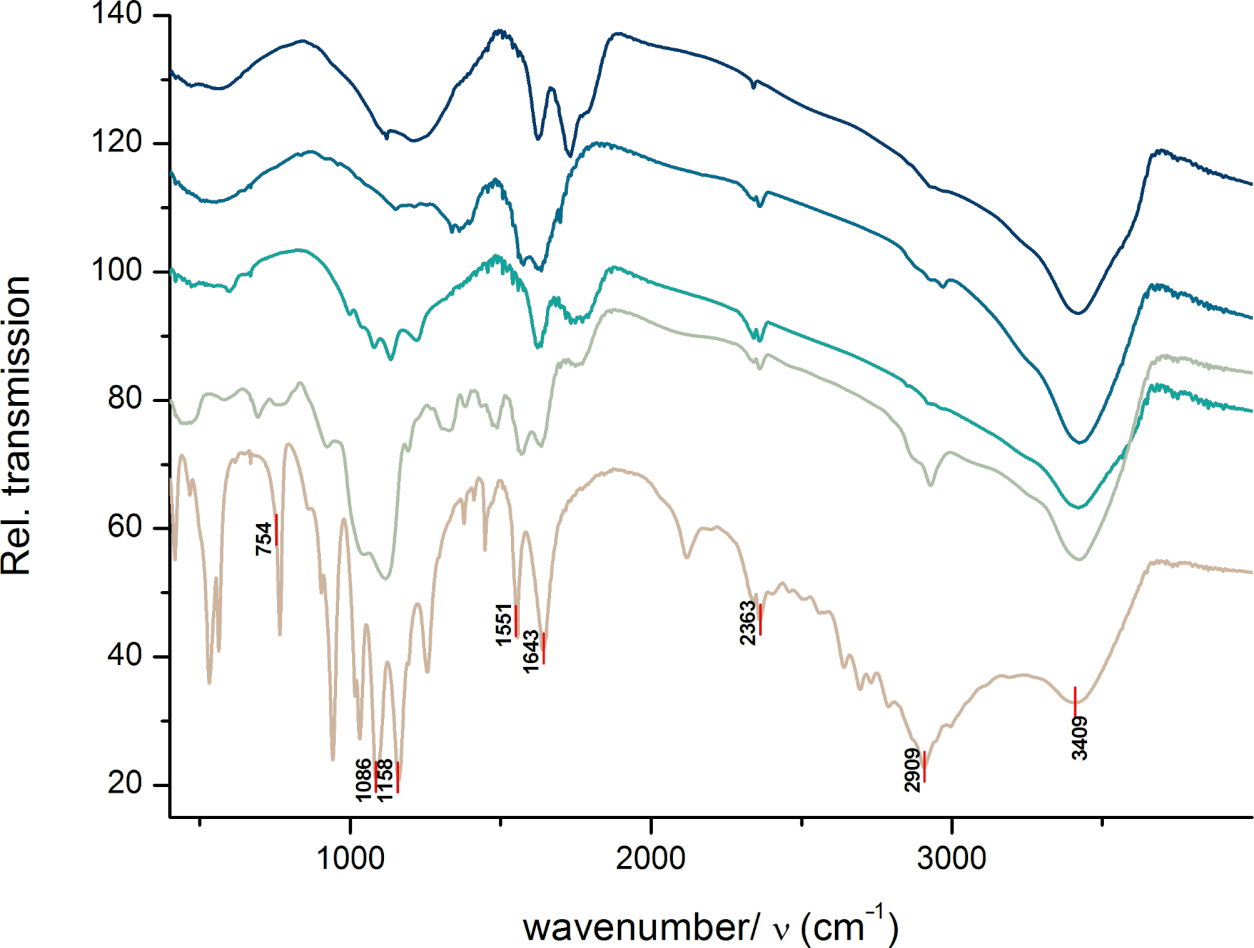
ATR-FTIR spectra of species involved in the synthesis process, from Scheme 1.

Figure 2 depicts the IR spectra of samples with phosphate groups coupled to the surface of the nanodiamond which use *O-*phosphorylethanolamine as main reagent. Figure 3 shows the infrared qualitative analysis of the efficiency of the synthesis achieved on the surface of DND, represented in Scheme 1.

Table S1 (please see the Supporting Information File 1) summarizes the characteristic absorption bands of the qualitative analysis from the synthesized species by IR spectroscopy [40–41]. The evaluation of the surface functionalization of DND can be proved by a qualitative analysis from the Figure 2 and Figure 3. The characteristic absorptions of some functional groups is directly related with the success of the synthesis, which are particularly observed on the assignments of the silane groups (1000 cm^−1^) in **4** and **5** and amino groups around 950 cm^−1^ and 1560 cm^−1^ in **4**, **5** and **6**. It is still possible to see some carboxylic groups on **4**, which may result from an incomplete oxidation process.

A positive signal of the integration of the phosphate group can be seen in **3** and **6**, at the wavenumber of 1090 cm^−1^ that is also present in the *O-*PEA.

In order to prove the covalent character of the formed bonds, physisorption tests were performed (see Supporting Information File 1). The tests demonstrated that no physical loading occurred on the surface of the nanodiamonds by the functionalizing agent, *O-*PEA.

### Immobilization of DND

Scanning electron microscopy (SEM) was used to characterize the surface morphology of titanium samples coated with previously functionalized nanodiamonds. DND before and after functionalization were used to evaluate the improvement on the interaction of DND with the titanium alloy surface.

**Figure 4:**
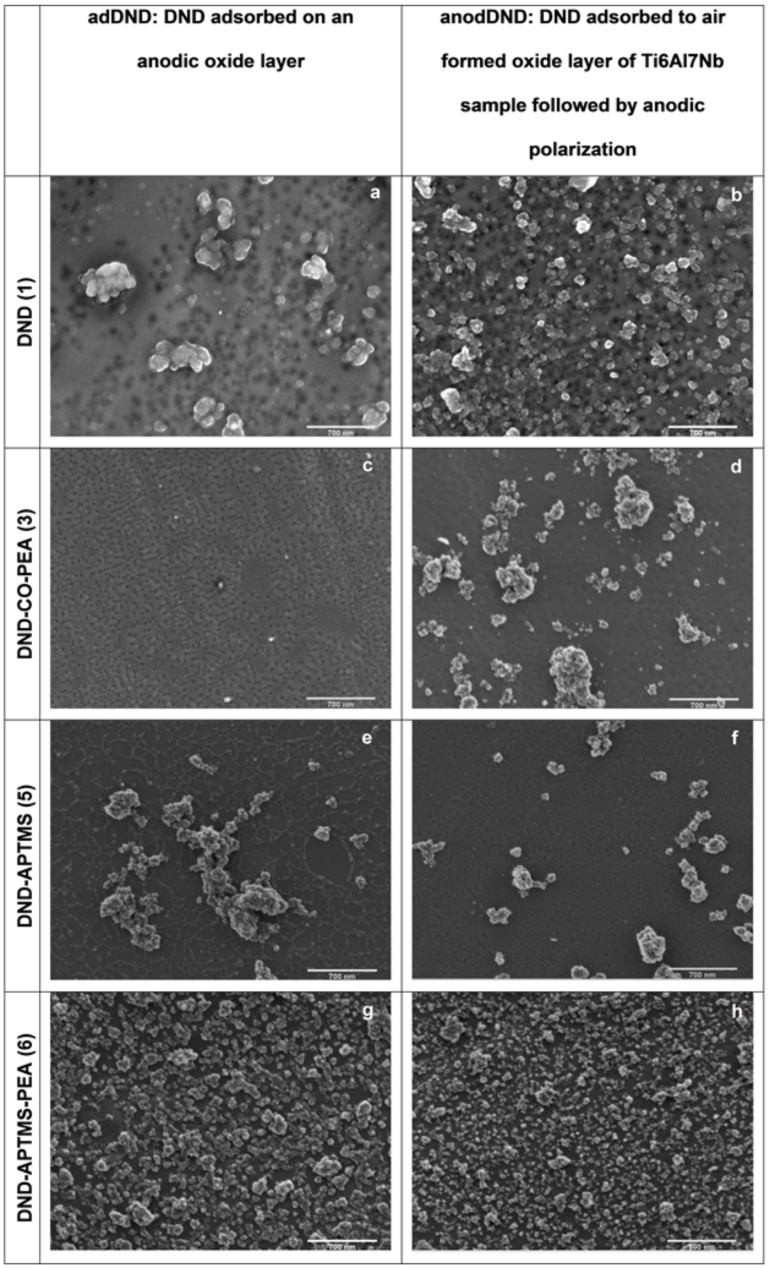
SEM images of titanium anodic oxide surface with: (a,b) unmodified DND; (c,d) DND-COOH functionalized with *O*-PEA (**3**); (e,f) silanized nanodiamonds with APTMS **5** and (g,h) silanized nanodiamonds functionalized with *O*-PEA **6**. Left column – adDND. DND adsorbed on an anodic oxide layer and right column – anodDND with adsorption of DND on air formed oxide layer of Ti sample followed by anodic polarization. Anodic polarization was consistently carried out at 60 V and 50 mA/cm^2^.

Figure 4 shows the topological information of all nanodiamond entities immobilized onto Ti6Al7Nb surfaces either by adsorption to preformed anodic oxide layers (adDND), or by adsorption to air formed oxide layers followed by growth of the oxide layer by anodic polarization (anodDND). Image g and h in which silanized DND functionalized with *O*-phosphorylethanolamine depicts more homogeneous distribution indicating better interaction with titanium alloy surface when compared to other entities. Moreover, the particle size and agglomeration are also effectively reduced after the functionalization with *O*-PEA (image h), compared to the unmodified DND (image b).

**Figure 5:**
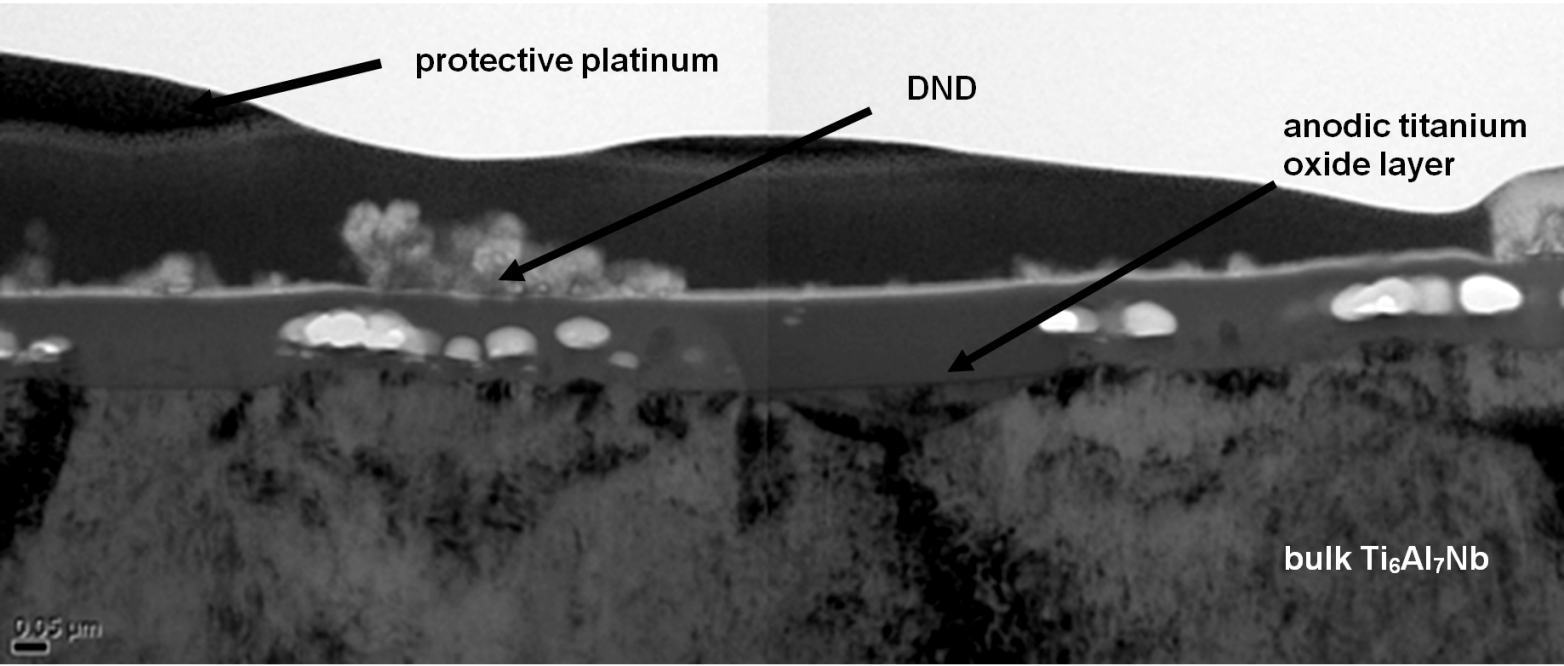
STEM image of immobilized aminosilanized DND **6** adsorbed to the air-formed passive layer of Ti_6_Al_7_Nb sample followed by anodic polarization (60 V, 50 mA/cm^2^).

STEM (scanning transmission electron microscopy) investigation of a FIB (focused ion beam) cut reveals a well-defined layer system as shown in Figure 5, for the immobilized aminosilanized DND **6** adsorbed to air formed oxide layer of Ti6Al7Nb followed by anodic polarization. The titanium based alloy substrate at the bottom is covered on top with an anodic oxide layer (thickness approx. 120 nm). An immobilization of **6** on titanium anodic oxide layer can clearly be seen, which was already indicated in the SEM images in Figure 4 for **6**. At this point, successful immobilization of **6** on the anodic oxide layer can be confirmed but with no signs of incorporation so far within the anodically grown oxide layer of the sample.

The initial interaction between all used entities (functionalized and unfunctionalized) of DND with the oxide layers on the titanium alloy surface is regulated by the pH of the electrolyte. Since the isoelectric point of these oxide layers lies between pH 4 and 4.5, the titanium dioxide surface will be negatively charged at the physiological pH of 7.4 [21].

Interactions between unmodified DND, and substrate are caused by hydrogen bridging and electrostatic affinity to positively charged amino groups. SEM results Figure 4 from unfunctionalized DND demonstrate a better interaction with the titanium oxide layer than carboxylated DND with *O*-PEA **3** and silanized DND **5**. This may be explained by the presence of several functional groups around the carbon core of the DND, as shown in Figure 1. These functional groups are originated in the purification treatment after the detonation process [28]. When in solution, the polarity of those functional groups may facilitate the formation of an aqueous suspension that was longer stable and translucent than the other suspensions prepared, although no longer stable than sample **6**. This functionalized sample also performed a homogenous suspension and was stable for 12 h. This fact establishes a strong correlation between the suspension stability and a well distributed surface adhesion of the related DND on the titanium oxide layer.

As an explanation for the homogenous immobilization of **6,** the authors believe the ability to form a stable suspension with **6** and the presence of phosphate anchor groups facilitates binding of finer nanodiamonds to the titanium oxide surface as seen in Figure 4, images g and h. Especially in the field of nanoparticles, entropic and surface effects, play a meaningful role that supports the electrostatic forces and possible covalent interaction, therefore the immobilization leads to binding of more and finer nanodiamonds (Figure 4g) and the incorporation increases the binding stability, leading to a meliorated surface distribution (Figure 4 h) of the small agglomerates of the functionalized nanodiamonds with *O*-PEA **6**, when compared to the all other tested samples. During the preparation of the nanodiamond suspension, silanized DND functionalized with *O*-phosphorylethanolamine revealed to be stable for 12 h. The strong phosphate attraction to titanium, even at neutral or weak basic pH, results in a firm adhesion of samples with phosphate moieties to the titanium dioxide layer [20,41]. Sample **6** possesses amino groups that build hydrogen bridges with the surface of the titanium alloy. The stable interaction observed is also achieved by coordinative binding of the oxygen in the phosphate group, which support the formation of monolayers on the titanium [42–43].

## Conclusion

Covalent functionalization of DND was carried out by different paths, with the main aim to couple phosphate groups. Successful DND functionalization was confirmed by infrared spectroscopy, depicting the presence of the attached functional groups at the DND surface. SEM results show better adhesion and homogeneous immobilization on the titanium alloy for the nanodiamonds functionalized with the phosphate group after the silanization process. These results indicate that the surface functionalization of DND is one important parameter for successful adsorption and immobilization on titanium alloy substrate. Another crucial parameter is to achieve a stable suspension of nanodiamonds. Sedimentation and agglomeration of several modified DND appeared to have a substantial influence on the success of the electrochemical immobilization. Silanized DND modified with *O*-phosphorylethanolamine **6** showed better dispersion in buffer compared to other samples and hence resulted in more homogeneous deposition during the electrochemical immobilization. We applied an electrochemical technique of anodic oxidation to get a stable immobilization of the particles during anodic oxide layer growth. A FIB-STEM tool was used to characterize the topology and layer system of titanium based alloy after immobilization of **6**.

In order to produce a stable suspension with the functionalized DND in different solvents and different acidic conditions, to optimize the electrochemical process, further experiments need to be performed. Further work is needed to describe in detail the adhesion mechanism of **6** on an anodic oxide layer. Stability and biocompatibility investigations of the achieved coatings will then follow.

## Experimental

### Chemicals

DND were purchased from Plasmachem GmbH (purity degree: 95%). All buffers where made from p.a. grade chemicals supplied by Merck KGaA and autoclaved before use. All other chemicals have been purchased with p.a. quality from Roth, Sigma-Aldrich and Fluka and were used without further purification.

### Carboxylated DND functionalized with *O*-phosphorylethanolamine

200 mg of carboxylated DND **2** were washed with 0.1 M 2-(*N*-morpholinoethanesulfonic acid) (MES) and resuspended in 4 mL of MES containg 6 mg of 1-ethyl-3-(3-dimethylaminopropyl)carbodiimide (EDC). The mixture was stirred for 20 min at room temperature. Afterwards, 25 mL of *O*-phosphorylethanolamine (8% aqueous) solution was added to the activated particles and the suspension was stirred for 2.5 h at room temperature. The sample **3** was rinsed with phosphate-buffered saline (PBS, pH 7.2) and dried under vacuum.

### Silanization of DND

1 g of hydroxylated DND **4** was mixed in a round bottom flask with 200 mg of 3-aminopropryltrimethoxisilane (APTMS) for 48 h at room temperature, while stirring. Afterwards, the solution was centrifuged to prepare the washing step with 40 mL of acetone for 24 h at 40 °C. The sample **5** was then dried under vacuum.

### Silanized DND functionalized with *O*-phosphorylethanolamine

200 mg of silanized DND **5**, 80 mg of *O*-phosphorylethanolamine, 35 mg of DMAP, 50 mg of EDC and 20 mL of dichloromethane (DCM), reacted under ice cooling for one hour; afterwards the slurry was stirred at room temperature for 65 h. At the end, the sample **6** was washed twice with acetone and twice with water and dried under vacuum.

### Preparation of titanium alloy samples

Sample disks of Ti_6_Al_7_Nb with a diameter of 15 mm × 2 mm height were used and were ground down to a grain size of P500. Sample etching in a mixed solution of 1 M nitric acid (HNO_3_) and 0.4 M hydrofluoric acid at room temperature was carried out for 2 min. This was followed by ultrasonic cleaning in sterile deionized water for 30 min.

### Suspension of functionalized DND

Dried powder of functionalized DND was used to form slurry or suspensions. For this purpose, 0.1 g of functionalized DND were mixed in 100 mL deionized water and ultrasonicated for 60 min.

### Electrolytic solution

A borate buffer of pH 7.12 prepared from 0.2 M boric acid, 0.05 M borax and 0.1 M Na_2_SO_4_ was used as an electrolyte. The pH of an equal mixture of buffer and nanodiamond suspension is set to be 7.42 with an electrical conductivity of 8.70 mS/cm.

### Immobilization of modified ND

For immobilization and incorporation experiments, a setup developed by Michael et al*.* [22] is used. It consists of a specially designed conical cell, made from acrylic glass, which mounts a titanium based alloy sample inside. Only 1.8 cm^2^ circular surface of sample is exposed to the electrolyte solution. A platinum wire is used as a counter electrode. Potential is measured against a saturated Ag/AgCl electrode inside a 3 M KCl solution connected to a conical glass cell via salt bridge. The salt bridge is formed by filling a capillary plastic tube with agarose gel (2 % w/v in 2 mol/L borate buffer, pH 7.4).

The experimental conical cell has a capacity of 3 mL. 1.5 mL of an aqueous slurry of modified DND is poured inside the cell, followed by an adsorption time of 20 min. After adsorption, 1.5 mL of borate buffer was added to the cell and anodic polarization was carried under galvanostatic mode of operation with 60 V as target potential and a current density of 50 mA/cm^2^. With 60 V of target potential, an anodic oxide layer of approximately 120 nm is expected to be formed. A potentiostat/galvanostat (Voltalab 4.0 Radiometer Analytical) was used for polarization.

### Adsorption on titanium anodic oxide layer

For adsorption experiments, anodic polarization was carried out initially forming an anodic oxide layer on the titanium alloy sample by adding borate buffer. Once an anodic oxide layer is formed, the buffer is removed and the sample is washed with deionized water for three times. This is followed by pouring 1.5 mL of aqueous DND slurry on anodically oxidized sample surface. After 20 min of adsorption time, samples are rinsed in deionized water.

### Characterization

The FTIR measurements were carried out with ALPHA's Platinum ATR from Bruker. The samples were measured in solid state, without previous preparation. Parameters were: 32 scans, 4 cm^−1^ resolution; wavelength range 4000–400 cm^−1^.

SEM measurements for topological characterization were performed with a Carl Zeiss 1500STES Gemini SEM. All titanium alloy samples with and without immobilized DND were first sputtered with carbon before observation under microscope.

For STEM investigation a Carl Zeiss LIBRA^®^ 200 was used. Focused ion beam lamella was cut every time to observe the layer structure of the titanium alloy. Sample preparation includes carbon coating followed by platinum deposition for sample protection.

## Supporting Information

File 1 Physisorption test.
