# Supplementary material for: Detonation nanodiamonds biofunctionalization and immobilization to titanium alloy surfaces as first steps towards medical application
Source: Beilstein J Org Chem. 2014 Nov 26;10:2765–73. doi: 10.3762/bjoc.10.293 (PMC4273212; doi:10.3762/bjoc.10.293)
Supplement: File 1 — Physisorption test. [file Beilstein_J_Org_Chem-10-2765-s001.pdf]

Supporting Information

for

Detonation nanodiamonds biofunctionalization and immobilization to titanium alloy surfaces as first steps towards medical application

Juliana P.L. Gonçalves[§,1], Afnan Q. Shaikh[§,1,2], Manuela Reitzig[1], Daria A. Kovalenko[1,2], Jan Michael[1,3], René Beutner[2], Gianaurelio Cuniberti[2], Dieter Scharnweber[2] and Jörg Opitz*,[1,2]

Address: [1]Inspection and Diagnosis Methods, Fraunhofer Institute for Ceramic Technologies and Systems –Materials Diagnostics, Maria-Reiche-Str. 2, 01109 Dresden, Germany; [2]Max Bergmann Center of Biomaterials MBC, Technische Universität Dresden, Budapester Str. 27, 01069 Dresden, Germany and [3]Chair of General Biochemistry, Technische Universität Dresden, Bergstr. 66, 01069 Dresden

Email: Dr. Jörg Opitz - joerg.opitz@ikts-md.fraunhofer.de

* Corresponding author

[§]both authors contributed equally

Physisorption Test

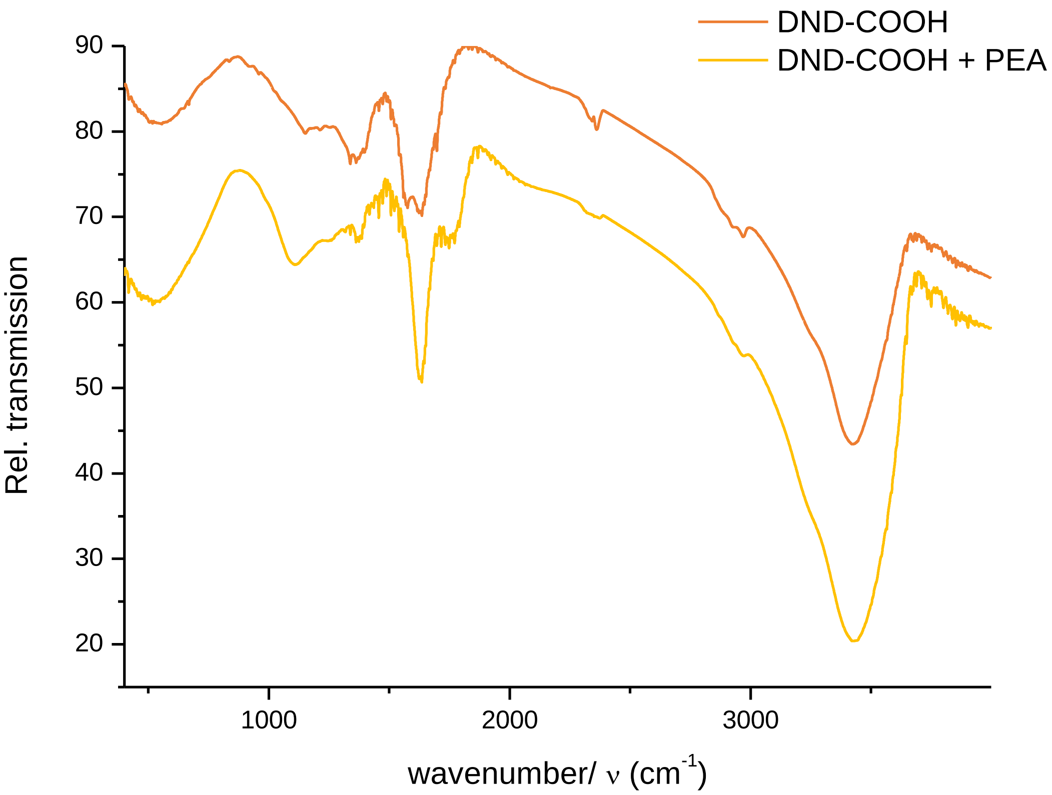

Figure S1: Physisorption test was carried out on carboxylic DND with *O*-PEA: An aqueous solution (10 mL) of 200 mg carboxilated DND **2** was allowed to react with *O*-PEA (8% aqueous solution) for 2.5 h, at room temperature. Both products were compared by FTIR. After the incubation time there is no signal form the *O*-PEA in the final product, indicating that no physisorption occurred.

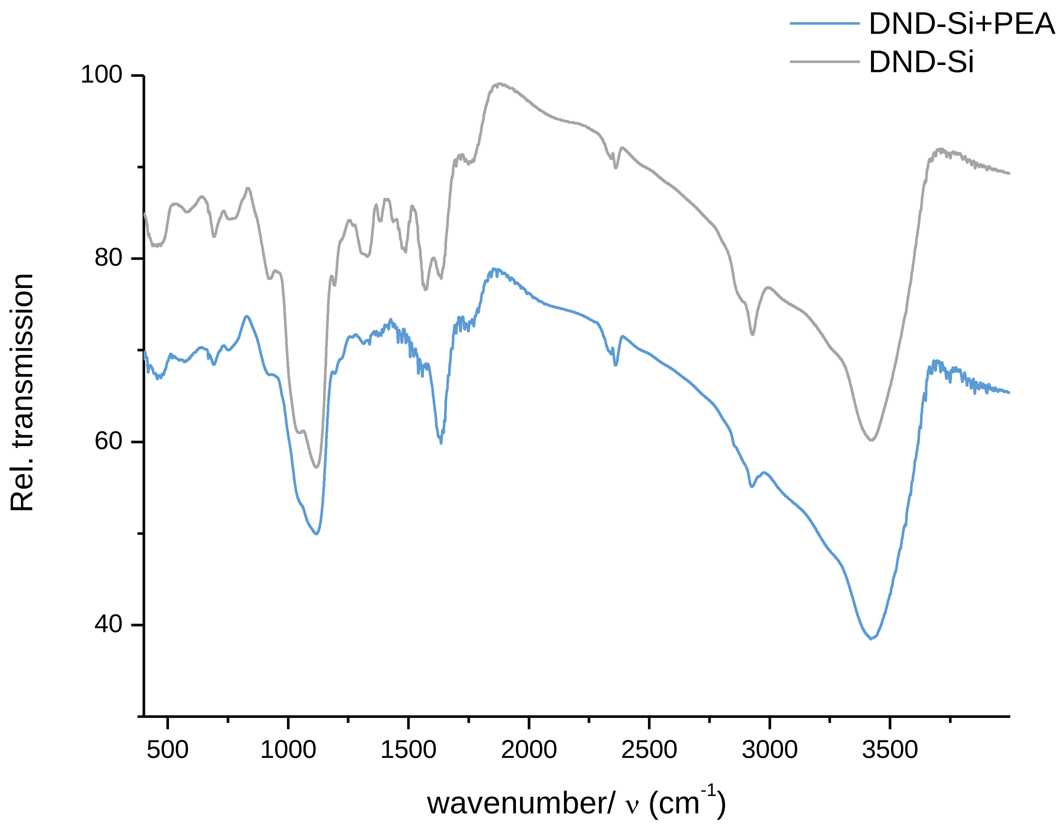

Figure S2: Physisorption test was carried out on silanized DND with *O*-PEA: An dichloromethane solution of 200 mg of silanized DND **5** and 80 mg of *O*-PEA, was allowed to react for 65 h, at room temperature. Both products were compared by FTIR. After the incubation time there is no signal from the *O*-PEA in the final product, indicating that no physisorption occurred.

Table S1: Characteristic absorption bands of species analyzed by infrared spectroscopy, Figure 2 and 3 [1,2].

DND<br><br>**1**	DND-COOH<br>**2**	DND-CO-PEA<br>**3**	DND-OH<br><br>**4**	DND-APTMS<br>**5**	DND-APTMS-PEA<br>**6**	*O*-PEA	*Characteristic absorption*
		515 cm^{-1}			535 cm^{-1}	525 cm^{-1}	
				690 cm^{-1}	570 cm^{-1}	565 cm^{-1}	
		750 cm^{-1}			775 cm^{-1}	760 cm^{-1}	
		930 cm^{-1}		925 cm^{-1}	940 cm^{-1}	943 cm^{-1}	δ_{NH2}
		1030 cm^{-1}		1050 cm^{-1}	1025 cm^{-1}	1025 cm^{-1}	Si-OR
		1106			1100 cm^{-1}	1085 cm^{-1}	Phosphate
1110 cm^{-1}	1150 cm^{-1}	1070	1170 - 1120 cm^{-1}	1120 cm^{-1}	1150 cm^{-1}	1155 cm^{-1}	ν_{C-O}
1220 cm^{-1}			1210 cm^{-1}		1250 cm^{-1}	1250 cm^{-1}	
	1340 cm^{-1}		1370 cm^{-1}	1320 cm^{-1}			$\delta_{C-O.H}$
	1570 cm	1500 cm^{-1}		1480 cm^{-1}	1560 cm^{-1}	1556 cm^{-1}	NH_2 scissoring
1620 cm^{-1}	1630 cm^{-1}		1630 cm^{-1}	1638 cm^{-1}	1638 cm^{-1}	1630 cm^{-1}	$\nu_{C=O}$
1735 cm^{-1}			1755 cm^{-1}			2110 cm^{-1}	O-H stretching
					2640 cm^{-1}	2640 cm^{-1}	
					2694 cm^{-1}	2900 cm^{-1}	$\nu_{Alkanes}$
2934 cm^{-1}	2970 cm^{-1}	2930 cm^{-1}	2920 cm^{-1}	2930 cm^{-1}	2910 cm^{-1}	2990 cm^{-1}	
3415 cm^{-1}	3420 cm^{-1}		3410 cm^{-1}	3420 cm^{-1}	3430 cm^{-1}		OH

References

1. Coates, Interpretation of Infrared Spectra, A Practical Approach. In: *J. Encycl. Anal. Chem.,* Ed, John Wiley & Sons: Chichester, 2000
2. Gong, W. *Int. J. Miner. Process.* **2001,** *63,* 147–165.
